# Assessing equity in preventing central line-associated bloodstream infections and surgical site infections in pediatric patients

**DOI:** 10.1017/ash.2024.502

**Published:** 2025-01-27

**Authors:** Xiaoyan Song, Deena Levey, Jenhao Jacob Cheng, Monica Monteon, Annette Lee, Nada Harik, Denice Cora-Bramble, Rahul K. Shah

**Affiliations:** 1 Office of Infection Control/Epidemiology, Children’s National Hospital, Washington, DC, USA; 2 The George Washington University, Washington, DC, USA; 3 Department of Quality & Patient Safety, Children’s National Hospital, Washington, DC, USA; 4 American Academy of Otolaryngology-Head & Neck Surgery, Alexandria, VA, USA

## Abstract

**Background:**

Central line-associated bloodstream infections (CLABSIs) and surgical site infections (SSIs) are major healthcare-associated infections that can be prevented by consistently applying evidence-based infection prevention practices.

**Objective:**

To assess equity in preventing CLABSIs and SSIs in pediatric patients at a free-standing pediatric hospital, where evidence-based infection prevention practices are consistently implemented.

**Methods:**

This observational study evaluated 2 cohorts of pediatric patients under 18 years. The CLABSI cohort included inpatients with a central line between 1/1/2016 and 12/31/2022, and the SSI cohort included patients undergoing colon, ventricular shunt, or spinal fusion surgeries between 1/1/2016 and 10/31/2022. The CLABSI rate per 1000 central line days and SSI rate per 100 surgeries were compared across different racial, ethnic, and gender groups.

**Results:**

In the CLABSI cohort of 8575 patients, encompassing 243,803 central line days, there were 156 CLABSIs. There was no statistical difference in CLABSI rate across race, ethnicity, and/or gender groups. The SSI cohort included 68 SSIs among 1710 patients who underwent 2230 procedures, including 714 colon, 749 ventricular shunt, and 767 spinal fusion procedures. The SSI rate was statistically higher in multiracial (9.9) and Asian (8.6) groups, compared to Caucasian (2.4) and Black (2.2) groups. A nested case-control study of the SSI cohort showed a higher SSI rate in Asians, compared to Caucasians (Odds Ratio: 3.3; 95% CI: 1.3–8.3).

**Conclusions:**

Equity in preventing CLABSIs is achievable through standardized central-line care. Further study is warranted to assess if improvement opportunities exist for achieving equity in preventing SSIs.

## Introduction

Modern healthcare in the United States continues to strive for high-quality and safe patient care for the best patient outcomes. However, emerging evidence suggests disparities in patient outcomes based on demographics such as race, gender, ethnicity, and language proficiency.^1^ Central line-associated bloodstream infections (CLABSIs) and surgical site infections (SSIs) are broadly recognized as key indicators of healthcare quality and safety.^2,3^ Both outcomes have been utilized to evaluate and identify gaps in the provision of equitable and inclusive care and the few studies published to date resulted in variable findings. A single-center retrospective cohort study found elevated CLABSI rates among pediatric patients self-identifying as Black and those using a non-English language for medical care. These disparities persisted after adjusting for relevant risk factors.^4^ A large cross-sectional study of adult patients undergoing colectomy or abdominal hysterectomy showed Black race was not associated with SSI in the colorectal or the hysterectomy groups.^5^ However, a study utilizing the American College of Surgeons National Surgical Quality Improvement Program database revealed disparities in SSI rates between White/Non-Hispanic and Black/African-American patients across surgical subspecialties,^6^ and another study using the same database showed an association with increased morbidity, mortality, and readmissions for Black patients across surgical procedures and specialties within 30-day post-surgery.^7^

Amid these disparate findings, there has been a lack of pediatric-specific studies assessing equity in care as related to SSI risk. Moreover, strategies to prevent CLABSI and SSI are distinctly different. Specifically, CLABSIs that are defined by the Centers for Disease Control and Prevention to measure healthcare quality and safety occur primarily during a patient’s hospitalization in a healthcare setting. Therefore, stringent training and adherence to evidence-based best practices among healthcare providers are crucial and inevitable steps to prevent CLABSIs. On the other hand, SSIs can occur within 90 days after surgery, including after a patient has been discharged and is at home. Therefore, in addition to excellent hospital care by healthcare providers, preventing SSIs may demand a stronger partnership with patients and their caregivers at home to prevent these infections. This is particularly true for pediatric patients, whose care relies greatly on parents and guardians.

Taking together, we designed this study to assess if a patient’s sociodemographic factors including race, gender, ethnicity, and preferred language affect CLABSI and SSI rates in pediatric patients. By comparing CLABSI and SSI with their respective relationship with patients’ sociodemographic factors, we aimed to understand whether these factors were associated with varying infection risk, in the context of increased caregiver participation in the care of a patient at home.

## Method

*Study Setting:* With the approval of the Institutional Review Board, the study was conducted at Children’s National Hospital (CNH), a free-standing pediatric facility located in Washington, D.C. serving patients in the greater metropolitan area. CNH has 323 beds for inpatient services that include level IV neonatal intensive care, pediatric intensive care, cardiovascular surgery intensive care, oncology, bone marrow and solid organ transplant, general pediatrics, and more. CNH also has 2 emergency departments and several outpatient primary and specialty clinics that serve pediatric children in Washington, D.C., and surrounding communities.

The Greater Washington region is home to 400 international associations and 1,000 internationally owned companies.^8^ In 2014, some 90,000 D.C. residents, or 15 percent of the population spoke more than a dozen different languages.^8^ During the study period, patients seen at CNH were 51% Black, 13% Caucasian, and 36% other races. Additionally, 69% of patients were not of Hispanic, Latino, or Spanish origin, 80% preferred English, and 19% preferred Spanish.

*Study Design and Patient Population:* The primary study design was a retrospective observational study including two cohorts of patients: the CLABSI cohort and the SSI cohort. The secondary study design was a nested case-control study using a subset of patients in the SSI cohort.

Patients in the CLABSI Cohort were selected from a line listing maintained by the Office of Infection Control for CLABSI surveillance and included patients who had a central line documented between January 1^st^, 2016 and December 31^st^, 2022 during an inpatient stay. Following CNH standard practice, nursing staff examined the central line including dressing status at the first encounter and hourly thereafter, and documented findings in the patient’s electronic medical record. Monthly, a computer program generated a list of patients with at least one documentation of a central line assessment. Infection Preventionists performed data validation using criteria established by the National Healthcare Safety Network (NHSN) and maintained the line listing for CLABSI surveillance. The Office of Infection Control also maintained a line listing of CLABSI cases that were identified by trained Infection Preventionists using NHSN definitions.

Patients in the SSI Cohort were selected from the SSI surveillance line listing maintained by the Office of Infection Control. Monthly, the Health Analytics team used Current Procedural Terminology codes defined by the NHSN to generate a list of patients undergoing ventricular shunt surgery, spinal fusion surgery, or colon surgery. Infection Preventionists then reviewed cases on the list to validate the procedure and identify SSIs using NHSN SSI definitions. The study included patients from the SSI surveillance line listing who had the surgery conducted between January 1^st^, 2016 and December 31^st^, 2022.

CNH electronic patient registry database was used to obtain demographic information including date of birth, self-reported gender, race, and ethnicity for patients in both the CLABSI cohort and the SSI cohort. Chart reviews were conducted for a nested case-control cohort that included SSI cases and randomly selected non-SSI cases (controls) to gather additional data including preferred language, interpreter use, and known SSI risk factors including age, wound classification, and American Society of Anesthesiologists (ASA) score. The cases and controls were matched only by surgery types.

*Data Analysis:* The analysis of the CLABSI cohort began with calculating the overall incidence rate (IR) per 1000 central-line days by dividing the number of CLABSIs by the number of central-line days. We then calculated the stratified IR by gender, race, and ethnicity. We employed Poisson regression to examine the statistical significance of variation in stratified incidence rates (IR). The 95% confidence interval (CI) was calculated to determine the statistical significance of differences in comparison groups.

The SSI cohort was analyzed by calculating the overall SSI rate per 100 procedures, obtained by dividing the number of SSIs by the total number of surgical procedures. Furthermore, we stratified the SSI rate by surgery type, gender, race, and ethnicity and examined the statistical significance of variation in these stratified rates using the chi-square test.

Mixed conditional logistic models were used to analyze the nested case-control data with the matching strata (surgery types) handled by a fixed effect and repeated patients by a random effect. One model was evaluated at a time for each of the patient demographics or risk factors using R software v4.3.1 (R Statistical Computing; Vienna, Austria) and mclogit package (v0.9.6; Elff 2022). Statistical significance was defined as *P*< 0.05 for two-sided tests.

## Results

The CLABSI cohort included 8575 patients with a total of 243,803 central line days. During these central line days, 156 CLABSI episodes were identified in 134 patients, resulting in an overall CLABSI rate of 0.6 per 1000 central line days. The CLABSI rate in male and female groups was 0.7 and 0.6 per 1000 central line days, respectively (Incidence Rate Ratio [IRR]: 0.9; 95% CI: 0.6 – 1.2). Compared to the CLABSI rate of 0.6 per 1000 central line days in the Caucasian group, CLABSI rates in all other groups, including Black (IR: 0.6 per 1000 central line days; IRR: 1.1; 95% CI: 0.7–1.7), Native Hawaiian, Pacific Islander, or Other (IR: 0.7; IRR: 1.3; 95% CI: 0.8–2.0), Asian, multiracial, or information unavailable (IR: 0.8; IRR: 1.5; 95% CI: 0.7 – 3.1) were comparable with no significant difference detected (Table 1). The CLABSI rate in Not Hispanic, Latino, or Spanish Origin group was 0.6 per 1000 central line days, which was lower than the Hispanic, Latino, or Spanish Origin group (0.8 per 1000 central line days). However, the difference in rates between the two groups was not statistically significant (IRR: 1.2; 95% CI: 0.9 to 1.8). (Table 1).

**Table 1.** Result of unadjusted analysis between central line-associated bloodstream infections (CLABSIs) and patient’s race, ethnicity, and gender

Race	CLABSI	Central Line Days	CLABSI Rate	IRR*	95% CI^
Asian	7	8713	0.8	1.5	0.6–3.5
Black	51	84273	0.6	1.1	0.7–1.9
Caucasian	32	58335	0.5	reference	–
Native Hawaiian, Pacific Islander, or Other Race	52	73690	0.7	1.3	0.8–2.1
Other (multiracial, declined or missing)	14	18792	0.7	1.5	0.7–2.6
**Ethnicity**					
Hispanic, Latino, or Spanish Origin	42	55018	0.8	1.2	0.9–1.8
Not Hispanic, Latino, or Spanish Origin	111	180274	0.6	reference	–
Other (multiple, declined, or missing)	3	8511	0.3	0.6	0.1–1.7
**Gender**					
Female	66	113216	0.6	0.8	0.6–1.2
Male	90	130535	0.7	reference	–
Missing information	0	52	0.0	0.0	–
**Total**	**156**	**243803**	**0.6**	–	–

* IRR: incidence rate ratio;

^ 95% CI: 95% confidence interval

The SSI cohort included 1710 patients who underwent 2230 procedures, including 714 colon, 749 ventricular shunt, and 767 spinal fusion procedures. Of these procedures, 68 SSIs were identified including 50 SSIs in colon surgeries, 7 SSIs in ventricular shunt surgeries, and 11 SSIs in spinal fusion surgeries. The overall SSI rate in the male and female groups were 3.8 and 2.5 per 100 procedures, respectively (IRR: 0.7; 95%: 0.4 – 1.1). The SSI rate was lowest in the Black (2.3 per 100 procedures), followed by Caucasians (2.5 per 100 procedures) and Other racial groups (3.2 per 100 procedures). (Table 2). The differences in the rates were not statistically significant. Asian, multiracial, or information unavailable groups had fewer than 100 procedures performed during the study period, resulting in high SSI rates ranging from 9.4 to 11.0 per 100 procedures in in Asian group and multiracial group, respectively. Both were statistically significantly higher than the Caucasian group. (Table 2).

**Table 2.** Unadjusted analysis between surgical site infections (SSIs) and patient’s race, ethnicity, and gender

Race Group	SSI	Non-SSI	SSI Rate	IRR*	95% CI^
Asian	6	64	8.6	3.5	1.4–8.1
Black	17	754	2.2	0.9	0.5–1.7
Caucasian	19	757	2.4	reference	–
Native Hawaiian, Pacific Islander, or Other Race	15	474	3.1	1.3	0.6–2.3
Other (multiracial, declined, or missing)	11	113	9.7	3.9	1.7–8.6
**Ethnicity**					
Hispanic, Latino, or Spanish Origin	13	387	3.3	1.1	0.5–1.8
Not Hispanic, Latino, or Spanish Origin	53	1732	3.0	reference	–
Other (multiple, declined, or missing)	2	43	3.8	1.5	0.2–5.8
**Gender**					
Female	28	1106	2.5	0.7	0.5–1.2
Male	40	1056	3.6	reference	–
**Total**	**68**	**2162**	**3.0**	–	

* IRR: incidence rate ratio;

^ 95% CI: 95% confidence interval

The nested case-control cohort included 68 SSI cases and 272 randomly selected non-SSI cases that were matched by surgery type only. There was no statistically significant difference in the odds for SSI related to age, gender, ethnicity, preferred language (Spanish vs. English), use of interpreters, refusal of use of interpreters in the non-English speaking group, ASA score, and wound class (Table 3). Compared to the Caucasian group, the odds for SSI were significantly higher in the Asian group (Odds Ratio: 3.3, 95% CI: 1.3 – 8.3) (Table 3).

**Table 3.** Results of nested case-control study assessing surgical site infections (SSIs) and patient’s race, ethnicity, and gender

Factor	Group	SSIs	Non-SSIs	OR*	95% CI^
Surgery	Colon	50	200	Reference	–
	Shunt	7	28	1.0	0.4–2.4
	Spine	11	44	1.0	0.4–2.0
Age (years)	<1	31	97	Reference	–
	1–5	8	50	0.5	0.2–1.1
	5–10	6	42	0.5	0.2–1.1
	10–18	19	69	0.9	0.4–1.6
	>18	4	14	0.9	0.2–2.8
Gender	Female	28	130	Reference	–
	Male	40	142	1.3	0.8–2.3
Race	Caucasian	19	95	Reference	–
	Black	17	80	1.1	0.5–2.2
	Other Race	14	65	1.1	0.5–2.3
	Multiracial	9	19	2.4	0.9–6.0
	Asian	6	5	5.8	1.6–23.0
Ethnicity	Not Hispanic	53	198	Reference	–
	Hispanic	13	70	0.7	0.3–1.3
	Other	2	4	1.9	0.2–10.8
Language	English	58	237	Reference	–
	Spanish	7	27	1.1	0.4–2.5
	Other	3	8	1.6	0.3–5.8
Use Interpreter	No	59	236	Reference	–
	Sometimes	8	25	1.3	0.5–2.9
	Yes	1	7	0.6	0.0–3.8
Refuse Interpreter	Yes	3	10	Reference	–
	No	65	262	0.8	0.2–3.8
ASA Score	1	4	24	Reference	–
	2	15	89	1.0	0.3–3.8
	3	42	137	1.8	0.6–6.5
	4	7	22	1.9	0.5–8.3
	E	18	43	2.4	0.8–9.4
Wound Class	Clean	32	104	Reference	–
	Clean-contaminated	34	164	0.7	0.4–1.2
	Contaminated	2	4	1.7	0.2–9.5

* OR: odds ratio;

^ 95% CI: 95% confidence interval

## Discussions

This is the largest pediatric-specific study to date that has used hospital-based Healthcare Associated Infection (HAI) surveillance data to evaluate equity in care in relation to the quality and safety of patient care as measured by CLABSIs and SSIs. As previously reported, hospital-based data can accurately identify HAIs such as CLABSIs, but administrative data cannot.^9^ Therefore, unlike a previous study that demonstrates higher CLABSI rates among Black and Hispanic children than their White peers using an administrative database,^10^ we find no disparity in CLABSI rates among pediatric patients of different genders, races, and ethnicities. Our achievement of equity in care is likely attributable to our institution-wide high-reliability central line care bundles and practices. In 2006, we implemented two central line care bundles (insertion and maintenance bundles) and two additional interventions (chlorhexidine scrub and chlorhexidine-impregnated sponges).^11,12^ In 2011, we added chlorhexidine daily bathing in the pediatric intensive care unit (ICU) and cardiovascular surgery ICU.^13^ In 2014, we standardized central line care by integrating unit-specific protocols into one institutional nursing practice guideline. In 2015, we incorporated the CLABSI prevention bundles introduced by the Solutions for Patient Safety network.^14^ In addition to the progressive introduction of evidence-based practices, we recognize the significance of implementation science in maintaining high reliability in central line care, not only among nursing staff but also among providers, such as residents and anesthesiologists who may need to access a central line. Our implementation bundle integrates ongoing competency-based education, the development of training videos, the provision of central line dressing change kits with step-by-step guides and necessary products, systematic auditing for compliance, and the provision of just-in-time training and review. We encourage patients’ family members and caregivers to participate in the patient’s care; however, they must undergo training before and be supervised when handling a patient’s central line. These standard central line care bundles have not only reduced the risks of CLABSIs but also ensured equity of care in patients with a central line. Consequently, the CLABSI rate that our institution achieved during the study period was 39% lower than the pooled incident rate reported by 110 children’s hospitals that submitted CLABSI data to NHSN during the same period.^15^

Of note, this is the first study to our knowledge that has included two types of HAIs in evaluating the relationship between HAI and demographic factors such as gender, race, and ethnicity. Similar to CLABSI prevention, we commit to providing standardized, evidence-based care to all patients undergoing surgery to ensure the best outcomes. Since 2016, we have implemented the SSI prevention bundle as recommended by Solutions for Patient Safety Network.^14^ We have achieved and maintained relatively low SSI rates in all three major procedures, colon, ventricular shunt, and spinal fusion, compared to the national pooled average of SSI rates across 287 pediatric hospitals.^16^ In addition, our SSI rates are uniformly low among Caucasian and Black groups and groups with different ethnicities, genders, preferred languages, and preferences for using interpreters. Our findings are different from previous studies showing higher SSI rates in Black groups.^17,18^

Combining both CLABSI and SSI cohorts, the only disparity that our study found is a higher SSI rate among the Asian group. The observed disparities may be due to the small sample size of Asian patients in our SSI cohort. It may also be due to that the Asian group recorded in the patient’s medical record is a diverse group composed of different subgroups as no more specific data was obtained about cultural identity. However, previous studies have shown that the Asian group is associated with higher SSI rates and higher refusal rates for oncological treatment.^19–21^ These findings highlight the need for targeted interventions in these minority groups for optimal patient outcomes.

We recognize that the study has two major weaknesses. One is that the retrospective nature of this cohort study using a hospital registry database as the data source for the demographic data is prone to known limitations.^22,23^ The other is that the study was conducted in one single center, therefore findings from this study may not be generalizable to all pediatric patient populations. Furthermore, our institution has aspired for high quality and safe care since 2009 when we began the Safety Transformation Initiative.^24–26^ In the “Journey to Zero,” the persistent adoption and enforcement of evidence-based standard care bundles and guidelines has empowered frontline healthcare providers to consistently deliver high-quality and safe care, effectively reducing disparities in patient outcomes to virtually zero. A major strength of this study lies in its considerable sample size. Furthermore, both cohorts of patients were sourced from the institution’s routine surveillance database, which is meticulously constructed and maintained to meet federal and local government requirements and comply with national standard definitions.

In conclusion, as evidenced by homogenous CLABSI rates across different racial, gender, and ethnical groups enrolled in this study, adherence to standard care practices is a powerful tool to prevent disparities in CLABSI risks. Further studies are warranted to identify strategies beyond standard care practices to achieve equity in preventing post-operative SSIs.
